# The diagnostic dilemma of adult-onset Still’s disease: a case report

**DOI:** 10.1097/MS9.0000000000002896

**Published:** 2025-01-21

**Authors:** Manoj Kumar Yadav, Aarati Rauniyar, Lalkiran Gharti Magar, Sangam Rouniyar, Bigyan Adhikari, Sandip Kumar Sah

**Affiliations:** a Department of Rheumatology, Nobel Medical College and Teaching Hospital, Biratnagar, Nepal; b Nobel Medical College and Teaching Hospital, Biratnagar, Nepal; c Nepalese Army Institute of Health Sciences, Sanobharyang, Kathmandu, Nepal

**Keywords:** adult-onset Still’s disease, arthritis, case report, fever of unknown origin, Yamaguchi criteria

## Abstract

**Introduction and importance:**

Adult-onset Still’s disease (AOSD) is a rare auto-inflammatory disorder, characterized by high-grade fever, arthritis, and a variety of systemic signs/symptoms. AOSD is very often misdiagnosed because of the overlapping clinical features, necessitating a thorough differential diagnosis, especially in cases of fever of unknown origin (FUO).

**Case presentation:**

A 55-year-old male with high-grade fever, myalgia, and arthralgia for the past 4 weeks. Yamaguchi criteria for AOSD met following an extensive evaluation. Laboratory findings showed leukocytosis with neutrophilic predominance, elevated ferritin levels, and mild abnormalities in liver function tests. The patient was started on intravenous corticosteroids, followed by oral corticosteroids in tapering dose of the drug and the introduction of methotrexate as a steroid-sparing agent (DMARDs).

**Clinical discussion:**

The case illustrates the diagnostic challenges associated with AOSD in older persons and the importance to consider this condition in the context of a FUO. The diagnosis of AOSD remains exclusive, yet effective management typically involves corticosteroids and DMARDs.

**Conclusion:**

AOSD, though rare, can occur uncommonly in older populations. This case highlights the need for awareness among clinicians to ensure early diagnosis and appropriate management, ultimately aiding in better outcomes of patient.

## Introduction

Adult-onset Still’s disease (AOSD) is a rare multisystemic auto-inflammatory disorder of unknown cause, affecting roughly around 1–10 people per million^[1,2]^. The peak age for the typical onset of AOSD is 15–25 and 36–46 years[3]. However, several case series have showed that AOSD can also affect elderly individuals^[2,3]^.

**Highlights**

- AOSD is a rare multisystemic auto-inflammatory condition of unknown cause, with an estimated prevalence of 1 to 10 cases per million.
- The disease usually affects young adults between the ages of 16 and 35; it is relatively less common in older populations. It is also increasingly considered an important cause of fever of unknown origin and should take into account while making differential diagnoses.
- Our patient presented with symptoms like spiking fever, arthralgia or arthritis, and leukocytosis. A diagnosis was made based on Yamaguchi’s classification criteria.
- The patient was treated with corticosteroids, in addition to nonsteroidal anti-inflammatory drugs (NSAIDs) and steroid-sparing agents such as methotrexate.

AOSD is typically characterized by high fever, arthritis, skin rash, and leukocytosis [an elevated white blood cell (WBC) counts of at least 10 000 cells/mm^3^ with neutrophils comprising 80% or more]^[3,4]^. Additional features like, a sore throat or pharyngitis, myalgia, enlarged lymph nodes or spleen, pleuritis or pericarditis, involvement of multiple organs, elevated liver enzyme levels, and other hematologic abnormalities may be present[4].

AOSD is a diagnosis of exclusion, and diagnosing it can be challenging due to the absence of specific diagnostic markers. Although Yamaguchi’s classification criteria are commonly used for diagnosis, but clinicians must first rule out infections, malignancies, and other rheumatic diseases[5]. Corticosteroids are usually the first line of treatment, followed by methotrexate, a disease-modifying antirheumatic drug (DMARD), and nonsteroidal anti-inflammatory drugs (NSAIDs)[6].

**Figure 1.**
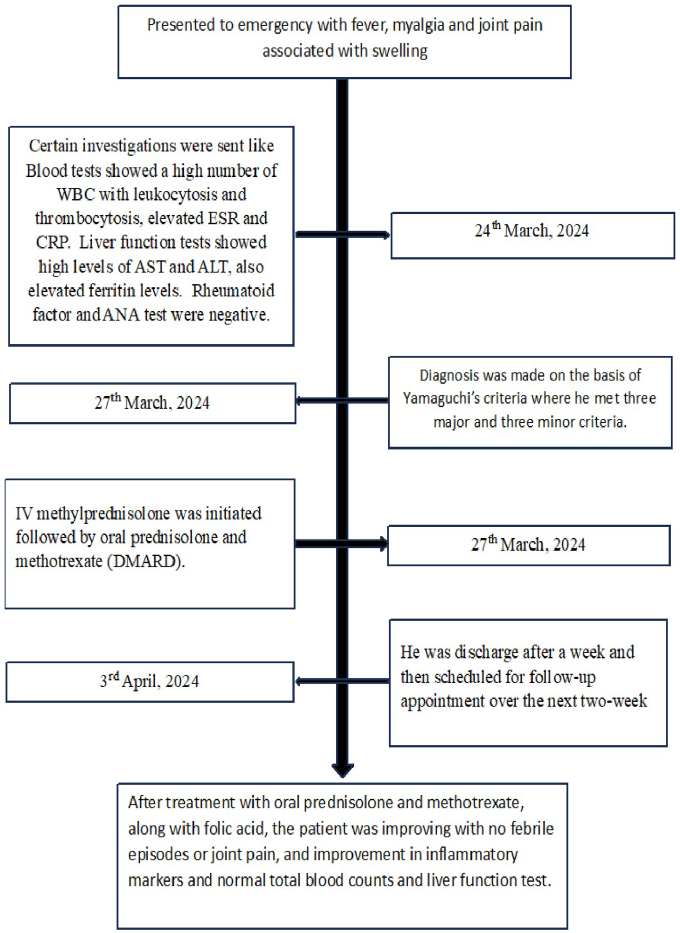
Timeline describing the course of disease in a patient.

We present a case of AOSD due to its rarity, and we emphasize its atypical age of onset and the diagnostic challenges faced by multiple healthcare institutions over the past 6 months. Our patient, a 55-year-old male, presented with a fever of unknown origin (FUO) and arthralgia, and has been diagnosed with AOSD using Yamaguchi’s criteria. He is currently on treatment. This case report has been reported in line with the SCARE Criteria[7]. Timeline describing the course of disease in a patient is shown in Figure 1.

## Patient information

A 55-year-old male, married, non-smoker, non-alcoholic, non-diabetic, and non-hypertensive was admitted to the Internal medicine ward via the emergency department. He had a history of recurring fever and joint pain over the past 6 months for which he visited multiple health institutes. He was prescribed NSAIDs which he usually discontinued after mild improvement in his pain and did not follow-up properly. Now he was presented, with a 4-week history of high-grade fever, myalgia, and pain in multiple joint associated with swelling. Fever was associated with chills and rigor and the maximum temperature documented was 39°C, he also complained of pain in joints involving small and large joints of both upper and lower limbs, associated with morning stiffness. There is no history of headache, cough, vomiting, chest pain, loss of consciousness, burning micturition, decreased appetite or weight loss. He had no other significant past medical history, was not allergic to any type of drugs and diets, and also had no significant history of similar illness in the family.

## Clinical findings

On physical examination, the patient was a moderately built, pale looking presenting with a fever of 39.0°C. His respiratory rate was 20 breaths per minute, pulse rate was 84 beats per minute and low blood pressure of 100/60 mmHg. There was also significant tenderness in all joints of the upper and lower limbs with swelling. Notably, there was no lymphadenopathy or splenomegaly, and other systemic examinations were unremarkable.

## Diagnostic assessment and interpretation

His initial blood and laboratory parameters revealed hemoglobin (Hb) of 11 g/dL, a WBC count of 22 800/mm^3^, and neutrophils comprising 80%. The platelet count was 556 000/mm^3^. A peripheral blood smear indicated microcytic hypochromic anemia (iron deficiency anemia), along with neutrophilic leukocytosis and mild thrombocytosis. Acute phase reactants were mildly elevated, with an erythrocyte sedimentation rate (ESR) of 45 mm/hour and C-reactive protein (CRP) at 134.1 mg/L, alongside elevated ferritin level. Liver function tests showed mild elevation, with alanine transaminase (ALT) at 52 U/L and alkaline phosphatase (ALP) at 326 U/L. Renal function tests were also mildly elevated, with urea at 62 mg/dL and creatinine at 1.9 mg/dL. Urine analysis, microscopic examination, lactate dehydrogenase, and creatine kinase levels were all normal. Additionally, both antinuclear antibody (ANA) and rheumatoid factor titers were negative (Table 1).

**Table 1:** Laboratory findings of the patient when he arrived at our hospital and a month after the treatment.

Tests	Initial report	Follow-up report (after 1 month)	Reference interval
**Complete blood count**			
White blood cells	22 800/mm^3^	10,700/mm^3^	4000–10 000/mm^3^
Platelets count	5 56 000/mm^3^	2 25 000/mm^3^	150 000–400 000/mm^3^
RBC count	4.42	4.9	4.0–5.9
Hemoglobin	11 g/dL	14.5 g/dL	13–17 g/dL
Hematocrit	32.8%	44.7%	40–54%
**Differential leucocyte count**			
Neutrophils	80%	74%	40–75%
Lymphocytes	10%	24%	20–40%
Monocytes	06%	01%	2–10%
Eosinophils	04%	01%	1–4%
Basophils	00%	00%	0–1%
**Biochemistry**			
ESR	45 mm/hour	36 mm/hour	0–20 mm/hour
CRP	134.1 mg/L	13.4 mg/L	<6 mg/L
Urea	62 mg/dL	–	15–40 mg/dL
Creatinine	1.9 mg/dL	1.0 mg/dL	0.6–1.3 mg/dL
**Liver function test**			
Total bilirubin	0.4 mg/dL	0.7 mg/dL	0.2–1.0 mg/dL
AST	45 U/L	21 U/L	14–63 U/L
ALT	52 U/L	29 U/L	15–37 U/L
ALP	326 U/L	86 U/L	46–116 U/L
LDH	268 U/L	–	225–450 U/L
Creatine kinase	40 U/L	–	<195 U/L
Total protein	7.4 g/dL	6.6 g/dL	6.4–8.2 g/dL
Albumin	3.0 g/dL	3.9 g/dL	3.4–5.0 g/dL
Ferritin	407 ng/mL	–	10–291 ng/mL
**Immunology**			
Rheumatoid factor	18 IU/mL	–	<20
Anti-DNA antibody	Negative	–	–

ALP, alkaline phosphatase; ALT, alanine transaminase; AST, aspartate aminotransferase; CRP, C-reactive protein; ESR, erythrocyte sedimentation rate; LDH, lactate dehydrogenase; RBC, red blood cell.

A chest X-ray, abdominal ultrasound, and pelvic CT scan were performed, all of which were normal except for the impression of fatty liver. Echocardiography results were also normal. The patient was subsequently referred to a rheumatology specialist for evaluation of his FUO in relation to his arthralgia and myalgia.

Based on his clinical presentation of daily high-grade fever, myalgia, arthralgia, significantly elevated leukocyte count with a predominance of neutrophils, and abnormal liver function tests, he met the Yamaguchi criteria after excluding infectious, autoimmune, and malignant diseases and was diagnosed with AOSD (Table 2).

**Table 2:** Yamaguchi criteria depicting common diagnostic criteria to help guide the diagnosis.

Major criteria	Minor criteria	Exclusion criteria
Fever > 102 F for >1 week	Sore throat	Infection
Arthralgia for >2 weeks	Lymphadenopathy	Malignancy
Typical rash	Hepatomegaly/splenomegaly	Other rheumatic diseases
WBC >10 000/mL	Abnormal liver function tests	
	Negative antinuclear antibody and rheumatoid factor	

WBC: white blood cell.

## Intervention

He was initiated on IV methylprednisolone at a dose of 250 mg per day for three days, followed by oral prednisolone at a dosage of 1 mg/kg (60 mg) in a tapering schedule and steroid-sparing drug (DMARDs), methotrexate at a dose of 10 mg per week, along with folic acid at a dosage of 5 mg per week.

## Follow-up and outcomes

During follow-up, he was improving, with no episodes of fever or joint pain. Repeat hematological investigations show normal total blood counts and normal liver function test, with improvement of inflammatory markers (ESR 35 mm/hour CRP 13.4 mg/dL). He was subsequently placed on a tapering schedule and is under regular follow-up.

## Discussion

In 1971, AOSD was first described by Bywaters and symptoms are similar to those of systemic juvenile idiopathic arthritis[6]. Although Still’s disease is relatively rare in adults, it is now recognized as a significant cause of FUO[8]. AOSD is a rare chronic inflammatory disease with a prevalence of around 0.16 occurrences per 100 000 cases with a bimodal age distribution between 15–25 years and 36–45 years[9]. It may be found in patients greater than 60 years of age[10]. Although the exact causes of AOSD is unknown, a number of factors, including genetic susceptibility, infectious triggers, immune dysfunction, and inflammatory activation, have been reported as potential contributions[11]. The most often accepted mechanism is auto-inflammation, in which a triggering factor that stimulates an immune response results in the release of pro-inflammatory cytokines leading to systemic inflammation[12].

AOSD is a diagnosis of exclusion and there are no particular diagnostic markers for AOSD, diagnosis can be difficult. Yamaguchi’s criteria are the most sensitive (96.3%) and specific (98.2%) of the recognized sets of diagnostic criteria (Table 2)^[5,13]^.

The major criteria include fever (higher than or equal to 39°C), joint pain, typical non pruritic salmon-colored rash, and leukocytosis (≥10 000 cells/mm^3^) with ≥ 80% neutrophils. Minor criteria include sore throat, lymphadenopathy, hepatomegaly or splenomegaly, abnormal liver function tests [including aspartate aminotransferase (AST), ALP, and ALT], and negative rheumatoid arthritis (RA) and ANA results. A patient must meet at least two major criteria and have no exclusion criteria in order to be diagnosed with AOSD. Clinicians also need to rule out infection, malignancy and other rheumatic illness. Although classification criteria are helpful for research, the Yamaguchi criteria show the highest sensitivity for diagnosis. The patient in our case met the major criteria of fever, arthralgia, and neutrophilic leukocytosis and positive minor criteria, including sore throat, abnormal liver function tests, and negative ANA and RA levels. He experienced arthralgia in multiple joints, most likely the interphalangeal joints. Our patient met three major features and three minor features of the Yamaguchi criteria.

Laboratory findings that support the diagnosis of AOSD include leukocytosis (especially neutrophils), anemia, elevated ferritin, CRP, ESR, and abnormal liver function tests (AST and ALT). Hyper-ferritinemia has been identified as a biomarker of AOSD among the laboratory abnormalities^[9,13,14]^.

AOSD has several severe complications, such as pericarditis, diffuse intravascular coagulation, liver failure, and respiratory failure^[9,15]^. Generally, the prognosis is better for patients with localized disease compared to those who experience severe complications^[15,16]^.

NSAIDs can be used to alleviate symptoms in the early stages, but the first-line treatment for AOSD is corticosteroids. Other treatment modalities in practice include DMARDs, tumor necrosis factor-alpha inhibitors, and interleukin inhibitors such as tocilizumab and anakinra^[14,17,18]^.

## Strengths and limitations

The case provides valuable clinical information by showing the application of Yamaguchi’s criteria and practical management techniques. Due to the rarity of AOSD, especially in older adults, this case contributes to the limited literature and encourages medical professionals to search for early diagnosis and treatment.

## Conclusion

AOSD is a rare disorder that is frequently misinterpreted due to its superimposed signs and symptoms. ASOD is an exclusive diagnosis, making it particularly challenging. This makes an extensive workup and multidisciplinary examination needed. The case illustrates the importance of considering AOSD in the differential diagnosis of FUO.

## Data Availability

All the findings are present within the manuscript.

## Patient’s perspective

The patient is well aware of his disease’s condition. He is satisfied with the treatment and has no objection in sharing about his condition after informing that his identification will remain confidential. He believes that it will help healthcare professionals to manage similar cases better.
